# Microglia in Alzheimer’s Disease: Activated, Dysfunctional or Degenerative

**DOI:** 10.3389/fnagi.2018.00140

**Published:** 2018-05-11

**Authors:** Victoria Navarro, Elisabeth Sanchez-Mejias, Sebastian Jimenez, Clara Muñoz-Castro, Raquel Sanchez-Varo, Jose C. Davila, Marisa Vizuete, Antonia Gutierrez, Javier Vitorica

**Affiliations:** ^1^Departamento Bioquímica y Biología Molecular, Facultad de Farmacia, Universidad de Sevilla, Seville, Spain; ^2^Instituto de Biomedicina de Sevilla (IBiS), Hospital Universitario Virgen del Rocío, CSIC, Universidad de Sevilla, Seville, Spain; ^3^Centro de Investigacion Biomedica en Red sobre Enfermedades Neurodegenerativas (CIBERNED), Madrid, Spain; ^4^Departamento Biologia Celular, Genetica y Fisiologia, Facultad de Ciencias, Instituto de Biomedicina de Malaga (IBIMA), Universidad de Málaga, Málaga, Spain

**Keywords:** Alzheimer disease, microglia, APP models, inflamation, Abeta plaques

## Abstract

Microglial activation has been considered a crucial player in the pathological process of multiple human neurodegenerative diseases. In some of these pathologies, such as Amyotrophic Lateral Sclerosis or Multiple Sclerosis, the immune system and microglial cells (as part of the cerebral immunity) play a central role. In other degenerative processes, such as Alzheimer’s disease (AD), the role of microglia is far to be elucidated. In this “mini-review” article, we briefly highlight our recent data comparing the microglial response between amyloidogenic transgenic models, such as APP/PS1 and AD patients. Since the AD pathology could display regional heterogeneity, we focus our work at the hippocampal formation. In APP based models a prominent microglial response is triggered around amyloid-beta (Aβ) plaques. These strongly activated microglial cells could drive the AD pathology and, in consequence, could be implicated in the neurodegenerative process observed in models. On the contrary, the microglial response in human samples is, at least, partial or attenuated. This patent difference could simply reflect the lower and probably slower Aβ production observed in human hippocampal samples, in comparison with models, or could reflect the consequence of a chronic long-standing microglial activation. Beside this differential response, we also observed microglial degeneration in Braak V–VI individuals that, indeed, could compromise their normal role of surveying the brain environment and respond to the damage. This microglial degeneration, particularly relevant at the dentate gyrus, might be mediated by the accumulation of toxic soluble phospho-tau species. The consequences of this probably deficient immunological protection, observed in AD patients, are unknown.

## Introduction

Alzheimer’s disease (AD) is characterized by complex molecular and cellular alterations, including the development of extracellular amyloid-beta (Aβ) deposits, intracellular aggregated phosphorylated tau, dystrophic neurites, loss of synapses and neurons, and a prominent gliosis. The reactive gliosis involves alterations in morphology and function of microglia and astrocytes (for recent reviews Heneka et al., 2015; Heppner et al., 2015; Calsolaro and Edison, 2016; Ransohoff, 2016; Cuello, 2017). In fact, the so-called neuroinflammative process is certainly a critical factor in the pathogenesis of multiple neurological disorders, such as inflammatory autoimmune diseases (e.g., multiple sclerosis). This inflammatory response is also clearly associated to the development of AD and microglia have recently emerged as crucial players in the pathogenesis of the sporadic forms (Heneka et al., 2015; Calsolaro and Edison, 2016; Ransohoff, 2016; Sarlus and Heneka, 2017) though it is still unclear whether a detrimental or protective but insufficient function contributes to disease. It has long been recognized that Aβ neuritic plaques are surrounded by activated microglial cells that could contribute to Aβ phagocytosis and/or compaction (Hickman et al., 2008; Frenkel et al., 2013). In fact, chronic microglial activation could improve the AD pathology reducing the Aβ levels in APP-based models (Michaud et al., 2013). However, the inflammatory response has also been associated with neurotoxic detrimental effects mediated by the release of proinflammatory cytokines/chemokines and neurotoxins (Kettenmann et al., 2011; Heneka et al., 2015; Heppner et al., 2015; Calsolaro and Edison, 2016). The implication of microglia in the AD pathology is also reinforced by human genome-wide association studies (GWAS). These GWAS analysis have identified multiple polymorphisms associated with the microglial immune response (Guerreiro et al., 2013; Cuyvers and Sleegers, 2016; Hansen et al., 2018). Within the different AD-associated genes, the microglial triggering receptor expressed in myeloid cells 2 (TREM2) gene seems to perform a pivotal role in the AD-associated immune response (Ulrich et al., 2017; Yeh et al., 2017; Hansen et al., 2018). TREM2 is a lipid and lipoprotein sensor that, through its adapter molecule DAP12, supports reactive microgliosis (Wang et al., 2015; Yeh et al., 2016). Furthermore, it has been recently demonstrated that TREM2, interacting with ApoE (the major genetic risk factor for AD), regulates the transcriptional activation of microglial cells (Krasemann et al., 2017). However, the role of TREM2-mediated microglial activation, or even the function of the microglial cells in the pathology of AD, is not elucidated (Ulrich et al., 2017).

Concerning to AD patients, the neuroinflammatory response is probably not exclusively detrimental or beneficial. An excessive microglial reaction could indeed be detrimental for the surrounding neurons or neuronal elements. However, the contrary, a deficient microglial response could also induce a similar detrimental effect on neurons. For instance, the absence of microglial response due to null mutations in TREM2 produces Nasu-Hakola disease, a rare neurodegenerative disease (Dardiotis et al., 2017). Therefore, the implication of the microglial response on the development of a neurodegenerative disease, such as AD, could be due to either an excessive or, on the contrary, a deficient microglial activation. In consequence, restoring microglial function may be a therapeutic option for AD treatment.

The achievement of effective therapies for AD requires modeling very specific aspects of the human pathology in the mouse models. Most of our knowledge about microglial activation in AD is so far based in studies using APP-based transgenic mice which robustly recapitulate the amyloid pathology but fail to develop neurofibrillary pathology. The failure of translating anti-inflammatory strategies into the clinical practice could be explained by a complex and differential glial response in AD models and patients. Here we provide an overview of the microglial response in amyloidogenic models and human *post-mortem* hippocampus with the aim of developing more predictive valuable animal models and gain success in clinical trials using neuroinflammatory targets.

## Decoding Microglial Activation in Mouse Models and Human Brains

**Figure 1:**
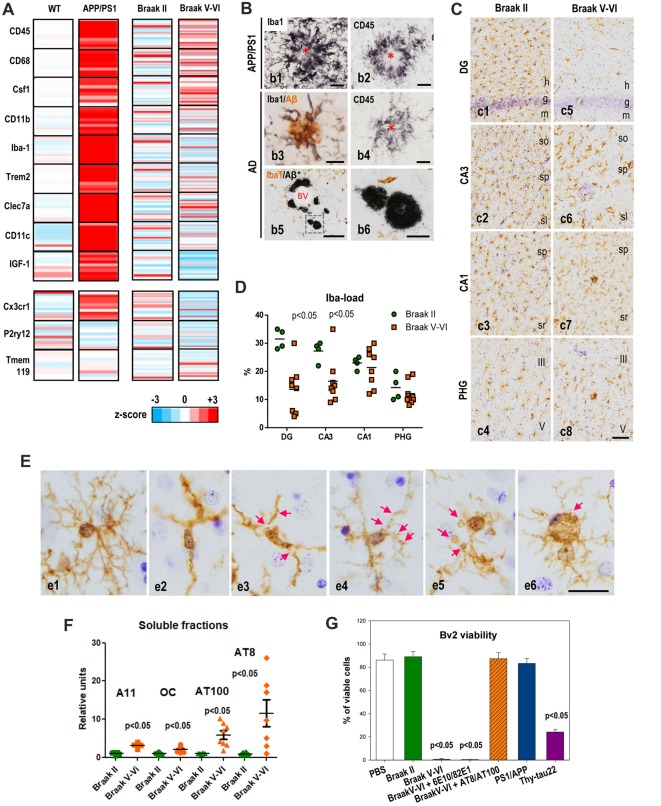
Microglial activation in APP/PS1 and microglial pathology in Alzheimer’s disease (AD) hippocampus. **(A)** Differential expression of selected microglial genes from hippocampus of 9- to 12-month-old APP/PS1 mice (age-matched wild-type (WT) as controls; *n* = 10 per genotype) and post-mortem human Braak V–VI samples (*n* = 28; age-matched non-demented BraaK II individuals as controls, *n* = 21) determined by qPCR analysis. Data were graphically plotted as *Z*-score and represented the deviation in the expression of a gene over the mean expression of this particular gene in the control populations (WT or Braak II). Each bar represented an individual murine or human sample. High expression is marked by the red color spectrum, low expression by blue colors. **(B)** Immunohistochemical detection of microglial cells around amyloid plaques in 12-month old APP/PS1 mice **(b1,b2)**, human AD brains staged as Braak IV **(b3,b4)** or Braak V–VI **(b5,b6)**. Amyloid plaques are surrounded by abundant activated Iba1-positive **(b1)** and CD45-positive **(b2)** microglia in the transgenic mice. Human plaques also displayed Iba1-positive and CD45-positive associated microglia at Braak IV stage, however the presence of amyloid deposits devoid of Iba1-positive microglia was often present in the hippocampus of Braak V–VI cases. Asterisks indicate amyloid plaques. BV, blood vessel. Scale bars **(b1–b4)** 20 μm; **(b)** 200 μm; **(b6)** 50 μm.** (C,D)** The Iba1-load quantitative analysis revealed a regional pattern with DG>CA3>CA1>parahippocampal gyrus (PHG) in Braak II (**C**,**c1–4**,**D**, green circles). In Braak V–VI individuals a significant (*p* < 0.05, Mann Whitney test) reduction of the microglial load was evidenced in the DG and CA3 regions (**D**, orange squares). **(E)** Compared to Braak II microglial cells (**e1** shows a non-activated healthy Iba1-positive microglial cell), Braak V–VI cases exhibit microglial cells with degenerative morphological features including deramification **(e2)**, fragmentation **(e3)**, beadings and spheroidal swellings **(e4,e5)** and dystrophies **(e6)** of the processes. h, hilar region; g, granular layer; m, molecular layer; so, stratum oriens; sp, stratum pyramidale; sl, stratum lucidum; sr, stratum radiatum. Scale bars, **(c1–8)** 100 μm; **(e1–6)** 20 μm. **(F)** Differential accumulation of soluble Aβ and phospho-tau forms in the hippocampus of human Braak II and Braak V–VI brains. Quantitative data from dot blots or western blots of soluble S1 fractions (extracellular/cytosolic) isolated from Braak II (*n* = 8) or Braak V–VI (*n* = 8) individuals. The relative abundance of A11 or OC positive Abeta oligomers, AT8 or AT100 phospho-tau proteins was determined by densitometry analysis. Significance was determined by Mann Whitney test. **(G)** Soluble phospho-tau toxicity on BV2 microglial cells. Microglia were incubated with S1 fractions isolated from Braak II, V–VI samples or 18-month-old APP/PS1 or 12-month-old Thy1-tau22 models. The toxicity was analyzed by flow cytometry and the percent of viable cells was showed. The Aβ or phospho-tau was immunodepleted from the Braak V–VI S1 fractions using 6E10 plus 82E1 or AT8 plus AT100 antibodies, respectively. The data are shown as the mean ± SD of six different experiments. Significance (indicated in the figure) was determined by ANOVA and Tukey *post hoc* test. Data were taken from Jimenez et al. (2008, 2014); Moreno-Gonzalez et al. (2009); Sanchez-Mejias et al. (2016); Baglietto-Vargas et al. (2017) and Gutierrez and Vitorica (2018).

APP-based transgenic mice displayed a considerable and progressive extracellular Aβ accumulation in hippocampus and cerebral cortex (Sasaguri et al., 2017). We and others (Jimenez et al., 2008; Heneka et al., 2015) have previously described that this extensive Aβ accumulation was accompanied by intense local microglial activation (see Figures 1A,B). More recently, the molecular signature of the microglial activation has been broadly studied using single-cell RNA-sequencing (Keren-Shaul et al., 2017). Using this powerful approach, genes associated to the microglial activation have been identified. In fact, these studies have clearly probed that microglial cells are activated and express a particular genetic program called “disease associated microglia (DAM)”. This DAM program includes the expression of multiple genes (such as Csf1, Clec7a, Igf-1), and some others associated to AD, like TREM2 or ApoE. The same active microglia down-regulate the “homeostatic” genes, such as Cx3cr1, P2ry12 or Tmem119 (Keren-Shaul et al., 2017). To investigate the microglial activation in our APP/PS1 model, we have directly determined the change in expression of some of these DAM and homeostatic genes from hippocampal samples. To briefly summarized our data, the change in the expression of microglial genes was displayed graphically as heat-maps (Figure 1A) by calculating the z-score between controls (wild-type mice, WT) and APP/PS1 samples (9- to 12-month-old; *n* = 10 per genotype). As expected, the levels of all these genes classified as part of the DAM program were noticeably and significantly (*p* < 0.05, *t-test)* induced. Furthermore, these activated microglial cells were located surrounding, and probably isolating, the Aβ plaques (Figures 1B,b1,b2). Therefore, as proposed previously, this microglial activation could exert a protective role in the AD pathology (Jimenez et al., 2008; Yeh et al., 2016; Yuan et al., 2016; Zhao et al., 2017). In this sense, it has been proposed that DAM phenotype could be neuroprotective (Keren-Shaul et al., 2017), although this role is actually under debate.

Concerning to the homeostatic genes, we have determined the expression of three of them, such as CX3CR1, P2ry12 and Tmem119 (see Figure 1A). As shown, in the APP/PS1 model, the expression of these particular genes was either not altered (P2ry12, Tmem119) or exhibited a small but significant (*p* < 0.05) increase (CX3CR1), compared with age-matched WT mice. As mentioned above, since the expression of these particular genes decreased upon “activation” (Keren-Shaul et al., 2017; Krasemann et al., 2017) these data were somehow conflictive. In fact, we have corroborated by *in vitro* experiments using primary microglial cells the decrease in these homeostatic genes after stimulation with either LPS or oligomeric Aβ (oAβ). Thus, the increase observed in some of these homeostatic genes could also reflect the proliferation of microglial cells in these models (Baglietto-Vargas et al., 2017). Microglia are self-renewal cells that, upon activation, could proliferate to develop an immune response (Askew et al., 2017; Tay et al., 2017). In fact, we also observed an increase in the expression of the mitotic marker Ki67 paralleled by increase in the BrdU incorporation on microglial cells (Baglietto-Vargas et al., 2017). Similarly, Füger et al. (2017) have reported a three-fold increase of the microglial proliferation in a different APP/PS1 model.

On the other hand, it has been classically assumed that the microglial response in AD patients could be similar to that observed in the amyloidogenic transgenic mice. Thus, we have also analyzed the microglial response in human hippocampus from non-demented Braak II controls (*n* = 21) and age-matched AD cases staged as Braak V–VI (*n* = 28). The expression of the different genes was calculated as z-score and showed individually in the graph (Figure 1A). Using this graphic approach, it is clear that the microglial reaction in AD samples differs obviously from that observed in the amyloidogenic model. An attenuated, rather than amplified, microglial response, was identified in the hippocampus of AD patients. As shown in Figure 1A; only few DAM genes were significantly (Mann Whitney test, *p* < 0.05) induced, such as CD45, CD68 and Csf1 in Braak V–VI samples, as compared with age-matched control individuals. Furthermore, this induction was clearly weakened as compared with APP/PS1 models. The apparently incomplete-activated microglial cells were principally surrounding the extracellular amyloid deposits (Figures 1B,b3,b4). On the other hand, the expression of homeostatic genes, such as Cx3cr1 and P2ry12, seemed to be significantly decreased in AD patients. We do not know whether this decrease in the expression reflects the partial microglial activation or, on the contrary, reflects a change in the microglial number.

Although microglial activation has been observed in cerebral regions with early and abundant extracellular Aβ deposits (Serrano-Pozo et al., 2013; Hamelin et al., 2016), our data clearly show that the microglial activation observed in the hippocampus of APP-based models undoubtedly differs both qualitative and quantitative from that observed in AD patients. This clear difference could reflect a different kinetic and/or magnitude of the Aβ accumulation. It is possible that the microglial response to the fast and extensive Aβ accumulation in transgenic models may resemble an acute response rather than a chronic and insidious pathology as observed in patients. On the other hand, and restricted to human samples, in Braak V–VI hippocampus we also observed neuritic plaques not surrounded by Iba1-positive microglial cells (Figures 1B,b5,b6). These “nude” amyloid plaques were not diffuse unreactive plaques. Thus, these data demonstrated the existence of substantial differences in the microglial response between APP-models and AD cases, at least at the hippocampal formation.

## Microglial Deterioration in the Hippocampus of AD Patients

As we pointed above, the microglial response in AD samples is quite dissimilar from that observed in APP-models. Furthermore, the existence of Aβ plaques with no surrounding/isolating Iba1-positive microglia led us to speculate with the existence of a pathological microglial process in AD patients. Thus, we analyzed using immunohistochemical staining the microglial response in AD cases (Braak V–VI) compared to controls (Braak II; Figure 1C). Using these experimental approaches, we first realized the existence of a regional microglial compartmentalization within the hippocampal formation. In Braak II control samples the parenchymal distribution of microglia cells, determined as Iba1-load, was significantly higher at the hilar region and CA3 than in CA1 or parahippocampal gyrus (PHG; Figures 1C,c1–4,D, green circles). The reasons and implications of this particular heterogeneity were actually unknown; however, it could reflect a different role or different immune-requirements associated to the particular connections between DG and CA3 regions. On the other hand (Figures 1C,c5–8,D), there was a clear and consistent (seven out of eight individuals tested) reduction in the microglial load (Iba1-immunopositive area) in Braak V–VI hippocampus. This reduction was not homogeneous for all regions analyzed. In fact, it was restricted to the hilar region of the DG and the CA3 subfield of the hippocampal formation. Other regions, such as CA1 and PHG were less affected, or unaffected, in the Braak V–VI samples. These data could indicate the existence of a possible microglial degenerative process restricted to DG and CA3 subfields. Thus, we focused our attention on the hilar region of the DG and further analyzed this putative microglial degeneration by determining: (1) the numerical density (cells/mm^3^) of Iba1-positive cells; (2) the parenchymal area covered by each microglial cell (microglial domain); and (3) the spatial distribution pattern of microglia (spatial covered). Interestingly, the numerical density of Iba1-positive cells was reduced in approximately 50% of the AD cases (Sanchez-Mejias et al., 2016). On the other hand, all tested samples showed clear modifications of the microglial morphology (Figures 1C,c1–4). In Braak II samples, microglial cells displayed a healthy morphology with highly ramified processes and were arranged with a regular spatial distribution covering, and probably protecting, most parenchymal space. This scenario was totally different in pathological Braak V–VI individuals. We observed a high and consistent reduction in both the microglial domain (area of surveillance of an individual cell) and in the spatial coverage of the hilar region of the DG (Sanchez-Mejias et al., 2016). In consequence, in AD hippocampus most of the parenchymal space of the hilar region was devoid on microglial protection.

This decrease was due to the existence of a clear pathological morphology of the microglial cells. As mentioned above, in Braak II samples, microglia showed a healthy morphology (Figures 1E,e1). On the contrary, Braak V–VI microglial cells displayed a shortened and less branched processes that usually were deformed, displaying cytoplasmic abnormalities (including spheroids) and even fragmentation (cytorrhexis; Figures 1E,e2–6). This microglial pathology in AD and even in Down syndrome brains has been previously reported (Lopes et al., 2008; Streit et al., 2009, 2014; Xue and Streit, 2011), however, it has never been detected in APP-based models (Figure 1; Jimenez et al., 2008; Heneka et al., 2015). This remarkable discrepancy between models and patients could also explain the patent differences in the expression of the DAM phenotype markers. As also mentioned, we also noted the existence of amyloid plaques without surrounding Iba1-positive microglial cells. If the microglial activation around Aβ plaques were indeed protective and constituted a preventive response by compacting plaques and isolating the putative toxic Aβ oligomers from the neuronal environment (Yuan et al., 2016), the existence of “nude” Aβ plaques in AD hippocampus could indeed increase the toxicity of these extracellular aggregates and drive neuronal damage and death.

## Soluble Phospho-Tau Is Responsible for the Microglial Degeneration in AD Brains

So far, we have demonstrated the existence of an evident pathological process in AD hippocampus affecting the microglial cells. To address which was the potential microglial toxic factor(s) in AD brains we examined whether soluble factors, such as oAβ or phosphorylated tau (phospho-tau) in Braak V–VI samples were the toxic agents (Sanchez-Mejias et al., 2016). In this sense, our previous work demonstrated that the hippocampus of APP-based models accumulated, in an age-dependent mode, soluble oAβ (Torres et al., 2012; Trujillo-Estrada et al., 2013; Jimenez et al., 2014). These soluble oAβ increased in aged APP-models and induced microglial activation in the inter-plaque areas (Jimenez et al., 2008). However, though the soluble oAβ also increased in Braak V–VI samples (tested using A11 or OC antibodies in dot-blots, Figure 1F), these oligomeric species were very scarce in Braak V–VI hippocampus (Jimenez et al., 2014). This apparent contradiction could be explained by the distinct development of AD pathology (lesions, course) between amyloidogenic models and AD samples. Models presented a high extracellular Aβ accumulation in both cortical areas and hippocampal complex since early ages (Trujillo-Estrada et al., 2014). However, human AD hippocampus, along with the entorhinal cortex, is one of the first regions to be affected by tau pathology and only, in very advanced stages, amyloid plaques are also present. In fact, the soluble fractions extracted from Braak V–VI hippocampus displayed relatively high content in AT100 and AT8 positive phospho-tau (Figure 1F) and relatively low oAβ levels.

We tested *in vitro*, using Bv2 cells or primary microglial cultures, whether soluble fractions (S1) derived from AD hippocampus were toxic (Figure 1G). The S1 fractions form Braak V–VI samples produced a clear reduction on the number of viable microglia, either Bv2 or primary cultures. This toxic effect was avoided by immunodepletion using a combination of AT8 plus AT100 antibodies whereas the same approach using 6E10 plus 82E1 monoclonal antibodies for Aβ produced absolutely no effect (Figure 1G). Thus, soluble phospho-tau seemed to be toxic for microglial cells *in vitro*. We further confirmed these results using soluble fractions extracted from aged APP/PS1 (18-month-old) or Thy-Tau22 (12-month-old) models. As expected, S1 fractions enriched in Aβ (APP/PS1; Jimenez et al., 2008) produced no effect whereas those containing phospho-tau (tau22) were toxic (Figure 1G). All these data demonstrated that, at least *in vitro*, soluble phospho-tau reduced the viability of microglial cells. This hypothesis was also supported by *in vivo* colocalization of dystrophic microglial cells with phospho-tau positive neuronal structures (Sanchez-Mejias et al., 2016).

**Figure 2:**
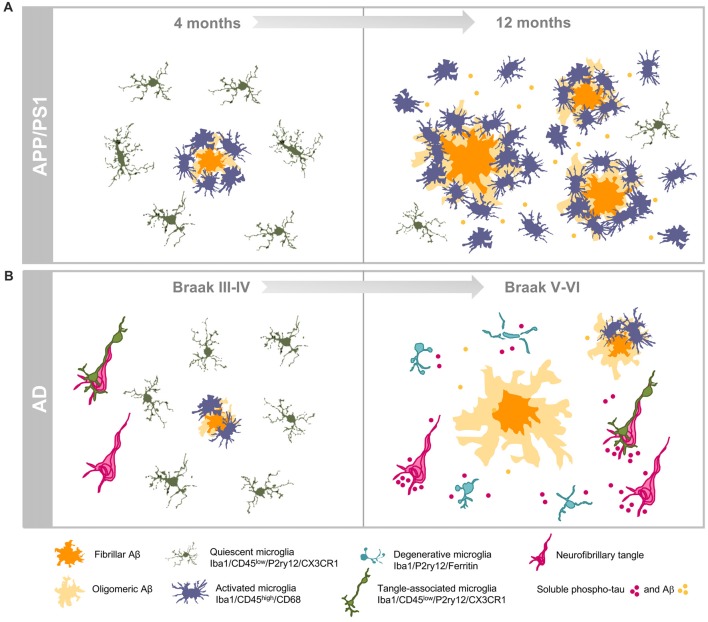
Dissimilar progressions of hippocampal microglial response in APP/PS1 mice and human brains. **(A)** Illustrate the age-dependent microglial activation in the amyloidogenic model from 4 (left) to 12 (right) months of age. Activated microglia characterized by morphological changes (hypertrophy of cell body and shortened processes) selectively cluster around amyloid plaques at early ages. Increased amyloid load with age is associated with expansion of the microglial activation. The age-dependent accumulation of soluble Aβ oligomers induces the activation of the interplaque microglia in aged transgenic mice. **(B)** Illustrate the microglial response during the course of AD pathology in the hippocampus of human brains. Amyloid pathology is a late event in the hippocampus while tau pathology shows a very early onset. In Braak III–IV hippocampus, some amyloid plaques are present and they are surrounded by activated microglia, however in late stage AD (Braak V–VI) microglial-bare plaques are usually found. The high content of phospho-tau forms (intra- and/or extracellular) induces the degeneration of microglial cells in human Braak V–VI hippocampus.

In sum, our work highlights relevant differences in the hippocampal inflammatory response between APP-transgenic mice and AD patients regarding microglial gene expression, morphology and survival (see Figure 2). In APP-models, the high and probably fast accumulation of extracellular Aβ produces a prominent microglial response. The activated microglial cells are predominantly located surrounding Aβ plaques, although active microglia are also identified in inter-plaque regions. While the role of the activated microglia could be neuroprotective, the strong microglial activation in APP models could also drive the AD pathology (Maphis et al., 2015; Olmos-Alonso et al., 2016; Spangenberg et al., 2016) and, in consequence, it could be implicated in the neurodegenerative process observed in APP-models. On the contrary, the microglial response in AD hippocampus is really mild. This patent difference could simple reflect the lower and probably slower Aβ production observed in human hippocampal samples, in comparison with models. However, and more relevant, in Braak V–VI samples there is a prominent microglial degenerative process that, indeed, could compromise their normal role of surveying the brain environment and respond to the damage. This microglial pathology, particularly relevant at the dentate gyrus of the hippocampal formation, might be mediated by the accumulation of toxic soluble phospho-tau species. The consequences of this probably deficient immunological protection due to the microglial degeneration, observed in AD patients, are unknown. Considering that microglial cells are implicated in multiple beneficial functions, such as Aβ phagocytosis, senile plaque compaction and limitation of Aβ toxicity and eliminating damaged neuron or neuronal debris (Yeh et al., 2016; Yuan et al., 2016; Ulland et al., 2017), a deficient microglial response could indeed aggravate the progression of AD pathology. In this sense, deficiencies on key genes for the microglial survival and/or proliferation (such as CSF1R or TREM2) are associated with rare hereditary neurodegenerative diseases, such as adult-onset leukoencephalopathy with axonal spheroids or Nasu-Hakola disease (respectively) (Paloneva et al., 2000, 2002; Chitu et al., 2016). In both diseases, the microglial response and, more relevant, the microglial survival seems to be compromised (Nataf et al., 2005; Satoh et al., 2011; Konno et al., 2014; Tada et al., 2016). On the other hand, missense mutations in TREM2 (such as R47H TREM2) produce an increases risk for late-onset AD (Guerreiro et al., 2013; Korvatska et al., 2015; Yeh et al., 2016) and a reduction in the number of microglial cells surrounding Aβ plaques in models. This decrease produced less compact and higher toxic plaques (Yeh et al., 2016; Yuan et al., 2016; Ulland et al., 2017). Therefore, a deficient rather than an exacerbated microglial response could be implicated in the development of sporadic AD.

## Author Contributions

JV and AG: drafting of the manuscript. ES-M, VN, JCD: design of figures. VN, ES-M, SJ, CM-C, RS-V, MV, JCD, AG and JV: critical revision of the manuscript for important intellectual content. All authors approved the manuscript in its final form.

## Conflict of Interest Statement

The authors declare that the research was conducted in the absence of any commercial or financial relationships that could be construed as a potential conflict of interest.
